# Efficacy of acupuncture versus sham acupuncture on generalized anxiety disorder: a meta-analysis of randomized controlled trials

**DOI:** 10.3389/fneur.2025.1682400

**Published:** 2025-11-12

**Authors:** Hongzhan Jiang, Ruiqing Ma, Yaxin Huang, Xuejing Li, Yufang Hao

**Affiliations:** School of Nursing, Beijing University of Chinese Medicine, Beijing, China

**Keywords:** acupuncture, generalized anxiety disorder, meta-analysis, randomized controlled trials, sham acupuncture

## Abstract

**Objective:**

This meta-analysis aimed to evaluate the specific efficacy of acupuncture compared to sham acupuncture in the treatment of generalized anxiety disorder (GAD), beyond nonspecific or placebo effects.

**Methods:**

We systematically searched PubMed, Web of Science, CNKI, WanFang, VIP, Cochrane Library, ClinicalTrials.gov, and EMBASE from inception to October 2025. Randomized controlled trials (RCTs) comparing acupuncture with sham acupuncture in adults diagnosed with GAD were included. Primary outcome was the Hamilton Anxiety Scale (HAMA), with secondary outcomes including Self-Rating Anxiety Scale (SAS), Generalized Anxiety Disorder 7-item scale (GAD-7), Pittsburgh Sleep Quality Index (PSQI), Self-Rating Depression Scale (SDS), cortisol (CORT), and adrenocorticotropic hormone (ACTH). Data were pooled using random- or fixed-effects models based on heterogeneity (*I*^2^). Risk of bias was assessed using the Cochrane tool, and evidence certainty was evaluated via GRADE.

**Results:**

Fourteen RCTs involving 968 participants were included. Acupuncture demonstrated significant reductions in HAMA [MD = −2.71, 95% CI (−4.17, −1.25), *p* = 0.0003], SAS [MD = −9.33, 95% CI (−16.29, −2.36), *p* = 0.009], GAD-7 [MD = −2.99, 95% CI (−5.52, −0.45), *p* = 0.02], PSQI [MD = −2.83, 95% CI (−5.37, −0.28), *p* = 0.03], and SDS [MD = −11.40, 95% CI (−19.89, −2.92), *p* = 0.008]. Small but significant effects were observed for CORT (SMD = −0.33, *p* = 0.007) and ACTH (MD = −3.18, *p* = 0.04). Heterogeneity was high for most outcomes. Evidence certainty was low to very low for patient-reported outcomes and moderate for biomarkers.

**Conclusion:**

Acupuncture is more effective than sham acupuncture in alleviating anxiety symptoms and improving sleep and mood in patients with GAD, though effect sizes are modest and evidence certainty varies. These findings support acupuncture as a potential non-pharmacological option for GAD, yet further high-quality trials are needed to standardize protocols and clarify mechanisms.

## 1 Introduction

Generalized anxiety disorder (GAD), characterized by persistent and excessive worry, is a prevalent mental health condition that imposes significant personal and societal burdens, with a global lifetime prevalence of 7.6% in some populations (1, 2). While first-line treatments such as selective serotonin reuptake inhibitors (SSRIs) and cognitive behavioral therapy (CBT) (3) are effective for many, a substantial proportion of patients experience inadequate symptom relief, adverse side effects (4), or poor adherence to long-term pharmacotherapy (5, 6). This gap in treatment efficacy and tolerability has spurred interest in complementary and alternative therapies, particularly acupuncture, which is increasingly recognized for its potential to alleviate anxiety symptoms with minimal side effects (7).

Recent systematic reviews and meta-analyses, including a 2022 study analyzing 27 randomized controlled trials (RCTs), reported that acupuncture is more effective than control interventions—including pharmacological treatments (e.g., SSRIs, benzodiazepines) and other active comparators such as Chinese herbal medicine—in reducing anxiety symptoms in GAD patients (8). However, because these control groups involve therapeutically active agents rather than inert placebos or sham acupuncture, such comparisons assess relative effectiveness, not specific efficacy. Consequently, the observed benefits cannot be attributed solely to acupuncture’s specific physiological mechanisms; they may reflect a combination of specific effects, contextual factors, and differential placebo responses between active interventions. This methodological limitation precludes a clear evaluation of whether acupuncture exerts effects beyond nonspecific or expectation-driven responses.

Sham-controlled trials are essential to isolate the biological and psychological mechanisms underlying acupuncture’s efficacy (9). A 2023 single-blinded RCT targeting perimenopausal women with GAD demonstrated that manual acupuncture (MA) outperformed sham acupuncture in reducing Hamilton Anxiety Scale (HAMA) scores (*p* < 0.001) and modulating adrenocorticotropic hormone (ACTH) levels. However, both groups showed improvements over time (10). This underscores the complexity of evaluating acupuncture’s true effect size, as placebo interventions may themselves exert psychophysiological effects (11). Despite such insights, existing meta-analyses have not comprehensively addressed sham-controlled designs, leaving the evidence base fragmented and inconclusive.

This meta-analysis aims to synthesize data from RCTs directly comparing acupuncture with sham acupuncture in GAD populations, addressing three critical gaps: (1) the scarcity of high-quality sham-controlled trials in prior reviews, (2) heterogeneity in outcome measures and intervention protocols, and (3) the need to quantify placebo-adjusted therapeutic effects. By elucidating acupuncture’s efficacy beyond nonspecific factors, this study seeks to inform clinical decision-making and refine guidelines for integrating acupuncture into evidence-based anxiety management strategies.

## 2 Methods

This systematic review was conducted in adherence with the guidelines outlined in the Cochrane Handbook for Systematic Reviews of Interventions (12) and was reported by the PRISMA statement (13). The review was also prospectively registered with the International Prospective Register of Systematic Reviews (PROSPERO) under Registration ID: CRD42024595883.

### 2.1 Inclusion criteria

Inclusion criteria: (1) peer-reviewed randomized controlled trials; (2) participants aged 18 years or older with a clinical diagnosis of generalized anxiety disorder; (3) comparison of acupuncture against sham acupuncture; (4) articles published in English or Chinese.

Exclusion criteria: (1) full texts cannot be obtained; (2) case series, duplicate publications, reviews, and conference proceedings; (3) literature with incomplete data.

### 2.2 Outcome measurements

#### 2.2.1 Primary outcomes: the Hamilton Anxiety Scale (HAMA)

Secondary outcomes: the Self-Rating Anxiety Scale (SAS), the Self-Rating Depression Scale (SDS), the Pittsburgh Sleep Quality Index (PSQI), the Generalized Anxiety Disorder 7-item scale (GAD-7), Cortisol (CORT), Adrenocorticotropic Hormone (ACTH).

### 2.3 Data sources

A comprehensive search was conducted across electronic databases: PubMed, Web of Science, China National Knowledge Infrastructure (CNKI), WanFang Data, and VIP Database (CQVIP), The Cochrane Library, ClinicalTrials.gov,^1^ and EMBASE, spanning all available years from database inception to October 2025. The search strategy utilized a combination of MeSH terms and free-text keywords. The detailed PubMed search strategy is provided in the Supplementary material S1.

### 2.4 Selection of studies

Two researchers (HJ and RM) independently executed the screening process. They first evaluated the titles and abstracts of all identified trials, subsequently obtaining and assessing the full text of pertinent publications. Any discrepancies were resolved through discussion until a consensus was reached. If a consensus could not be reached, a third reviewer (YaH) was consulted for arbitration.

### 2.5 Data extraction

Independent extraction of data from each study was performed by two reviewers (HJ and RM) utilizing a pre-defined and piloted data extraction form in Microsoft Excel. The reviewers worked independently and were blinded to each other’s results initially. The extracted information included study characteristics (authorship, year of publication, design, sample size), participant demographic details, intervention specifics (type of acupuncture, frequency and duration of sessions, selection of acupoints), details of the sham procedure, outcome measures (mean and standard deviation (SD) of scores before and after intervention), and funding sources. After independent extraction, the two datasets were cross-checked. Any inconsistencies were identified and resolved by referring back to the original publication. If necessary, a third reviewer (XL) was involved to achieve a final consensus. For studies with missing or incomplete data, we attempted to contact the corresponding authors via email to request the required information.

### 2.6 Quality assessment

The methodological quality of the included studies was assessed independently by two reviewers (HJ and RM) using the Cochrane Risk of Bias Tool (14). The reviewers evaluated the following domains: random sequence generation (selection bias), allocation concealment (selection bias), blinding of participants and personnel (performance bias), blinding of outcome assessment (detection bias), incomplete outcome data (attrition bias), selective reporting (reporting bias), and other potential sources of bias. The two reviewers conducted the assessments independently and without consultation. For each domain, judgments were assigned as ‘Low risk’, ‘High risk’, or ‘Unclear risk’ of bias, along with supporting information from the study publications. After the initial assessment, they compared their judgments. The certainty of the evidence was graded using the GRADE (Grading of Recommendations Assessment, Development, and Evaluation) according to (GRADE handbook). Any discrepancies were resolved through discussion. If a consensus could not be reached, a third reviewer (YFH) made the final decision, ensuring a consistent and unbiased appraisal.

### 2.7 Statistical analysis

Statistical analyses were performed using RevMan 5.4.1 and Stata 12.0. Continuous outcomes were analyzed via mean differences (MD) with 95% confidence intervals. Standardized mean differences (SMDs) with 95% CIs were calculated for studies using different outcome scales. Heterogeneity was quantified through Cochran’s *Q* test and *I*^2^ statistics, with *I*^2^ > 50% indicating substantial heterogeneity warranting a random-effects model; otherwise, a fixed-effects model was applied. To test the robustness of the meta-analysis results, a sensitivity analysis was performed using the leave-one-out method. This was done by systematically removing each included study one at a time and recalculating the pooled effect size for the remaining studies. This process allows for the identification of any single study that disproportionately influences the overall results. The results of this sensitivity analysis were synthesized and presented using forest plots generated in Stata 12.0.

### 2.8 Assessment of reporting biases

A funnel plot was employed to evaluate publication bias whenever the number of studies included in a meta-analysis exceeded 10. We additionally conducted statistical tests for publication bias using Egger’s linear regression test and Begg’s rank correlation test. These analyses were performed using Stata 12.0. A *p*-value of less than 0.05 in either test was considered to indicate potential statistical significance of publication bias.

## 3 Results

### 3.1 Literature screening

**Figure 1:**
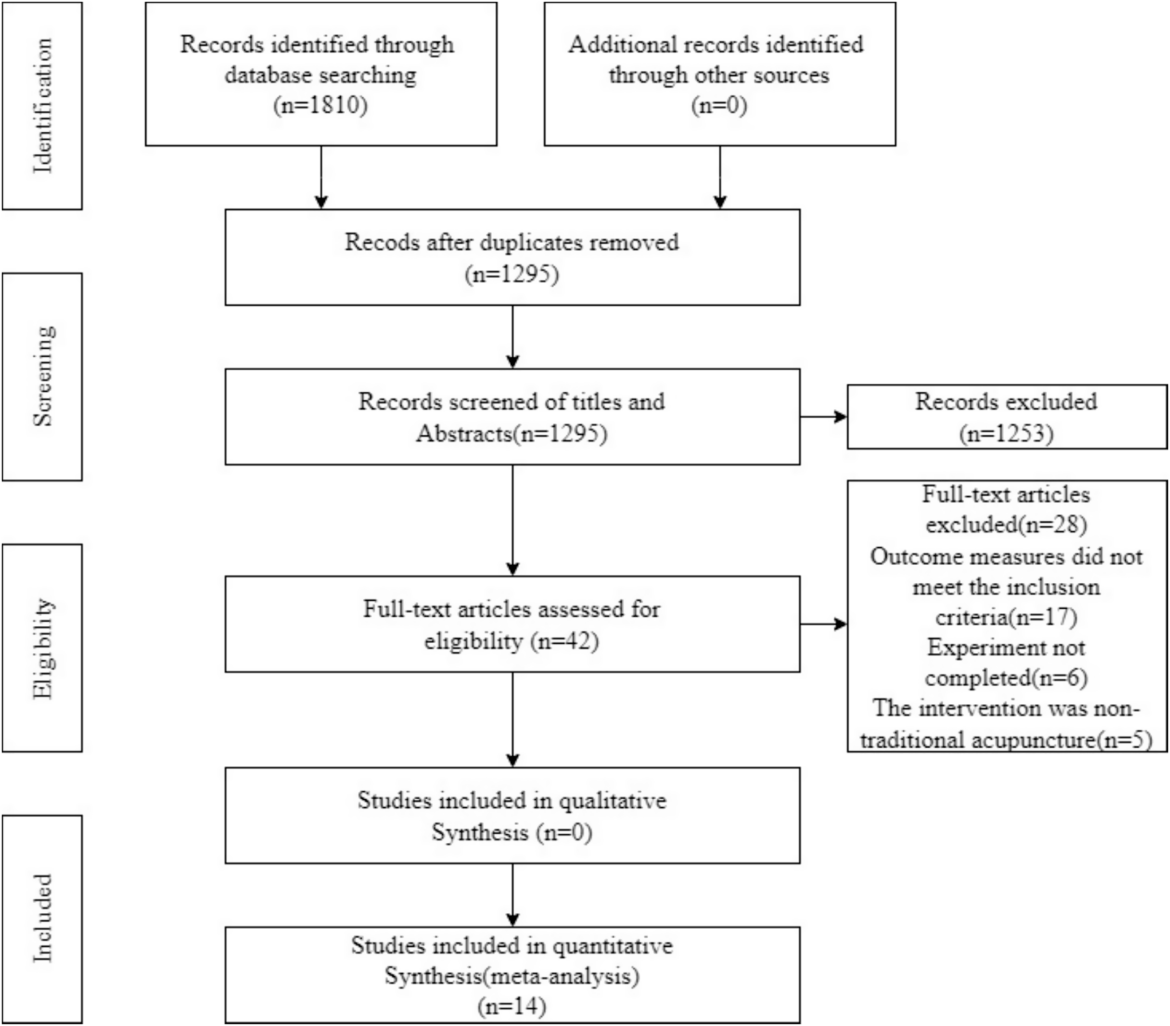
Search flowchart.

The initial literature search yielded 1810 references. After removing duplicates, 1,295 articles were evaluated. Of these, 1,253 were excluded based on title and abstract review. An additional 42 references were scrutinized in detail, with 28 being excluded. 14 studies were finally selected in this review (Figure 1).

### 3.2 Characteristics of studies

The 14 trials (10, 15–27) had 968 participants in total. All trials were conducted in China. The sample size of studies ranged from 44 to 90. Treatment durations spanned 2–8 weeks, with follow-up periods ranging from 1 month to 42 weeks. Acupuncture techniques included real acupuncture, regulating spirit acupuncture, ghost point acupuncture, electroacupuncture, abdominal acupuncture combined with herbal medicine, and intradermal needle therapy. Control groups consisted of sham acupuncture (non-insertive/blunt needles without Deqi sensation), sham acupuncture (non-penetrative), and sham needle devices. Common outcomes measured included HAMA, GAD-7, PSQI, CORT/ACTH levels (Table 1).

**Table 1:** Characteristics of studies.

No	Study	Country	Participants	Treatment duration	Intervention	Control	Sample	Outcomes
1	Liu et al. (10)	China	Perimenopausal women with generalized anxiety disorder (GAD), 45–55 years old	Treatment duration: 4 weeks; follow-up: 3-month period	Real acupuncture, 4-week, 12-session treatment	Sham acupuncture; by blunt tipped placebo needles that were not inserted into the skin and did not stimulate a Deqi sensation	88	HAMA, GAD-7, PQSI, CORT, ACTH, Adverse events
2	Gol et al. (23)	Iran	Patients with anxiety disorders, 18–60 years old	Treatment duration: 4 weeks	Acupuncture, three sessions per week for 4 weeks	Sham acupuncture: the placebo needles applied at real acupuncture points while they did not penetrate the skin and they were pushed into the handle	70	STAI, CORT
3	Liu (20)	China	Perimenopausal women with generalized anxiety disorder, 45–55 years old	Treatment duration: 4 weeks	Regulating spirit acupuncture, three times per week	Sham acupuncture; use the flat-headed acupuncture needle, without puncturing the skin, do not induce the feeling of Deqi	62	HAMA, GAD-7, PSQI
4	Huang et al. (22)	China	Patients with generalized anxiety disorder, 18 ~ 60 years old	Treatment duration: 2 weeks	The Ghost point acupuncture, after the first acupuncture, acupuncture treatment was performed every 1 day, 4 times a week, a total of 8 times	Sham acupuncture; used a special comfort needle. The needle does not puncture the surface of the skin	60	HAMA, Amplitude of the P300
5	Zhang et al. (18)	China	Patients with insomnia;18–65 years old	Treatment duration: 2 weeks; follow-up: 1 month	Active acupuncture; performed every 10 min to achieve a Deqi feeling	Sham acupuncture; received non-insertive acupuncture using the sham needle supported by the park device, could not penetrate the skin	90	PSQI, Sleep rate, SAS, SDS, Adverse events
6	Zhang et al. (17)	China	Insomnia patients with emotional disorders, 18–65 years old	Treatment duration: 2 weeks; follow-up: at 6, 18, and 42 weeks	Active acupuncture; the acupoints were artificially stimulated every 15 min, each acupoint was 5 s. The treatment was continued for five consecutive days, followed by 2 days of rest	Sham acupuncture; used the PSD combined with a blunt needle, did not experience Deqi	90	PSQI, SAS, SDS, Adverse events
7	Liu et al. (21)	China	Chronic insomnia patient, 18–70 years old	Treatment duration: 4 weeks; follow-up: 3 months	Acupuncture, three times per week	Sham acupuncture: needles were inserted 1 or 2 mm into the acupoints without manual stimulation and Deqi	56	Clinical improvement, HAMA, PSQI, CORT, HAMD, Adverse events
8	Zhang et al. (16)	China	Menstrual migraine without aura, 18–50 years old	Treatment duration: 27 sessions;	Acupuncture; 1 week before menses, once every other day for a total of three treatments	Sham acupuncture (no Deqi sensation)	44	Clinical improvement, SAS, SDS
9	Wang et al. (19)	China	Menopausal women with mood disorder, 41–60 years old	Treatment duration: 8 weeks; follow-up: 4 weeks	Abdominal acupuncture + Chinese herbal medicine; once a day for the first 3 days and subsequently once every 3 days	Sham acupuncture+ Chinese herbal medicine; short plastic needle sheaths containing no needles were tapped against the skin of the acupoints	63	Clinical improvement, SAS, SDS, Adverse events
10	Zhao et al. (15)	China	Perimenopausal women with comorbid depression and insomnia, 45–55 years old	Treatment duration: 8 weeks; follow-up: 16 weeks	Real-Acupuncture, three sessions per week for the first 3 weeks, two sessions per week for the next 3 weeks, and one session per week for the final 2 weeks	Sham-acupuncture: shallowly inserted into each acupoint thus avoiding any De-qi sensation	70	Clinical improvement, HAMA, PSQI, HAMD, adverse events
11	Fan et al. (24)	China	Parkinson’s patients with anxiety, 35–80 years old	Treatment duration: 8 weeks; follow-up: 8 weeks	Real-acupuncture + clinical monitoring + anti-Parkinson drugs, three times per week	Sham-acupuncture + clinical monitoring + anti-Parkinson drugs; underwent a non-insertion procedure applied at the acupoints	64	Clinical improvement, HAMA, CORT, ACTH, adverse events
12	Deng et al. (25)	China	Patients with subthreshold depression, 18–40 years old	Treatment duration: 6 weeks; follow-up: 1 months	Intradermal needle therapy, keep needles for 2 days, and treat twice a week	Sham needle; the tip of the needle does not penetrate the skin	78	Clinical improvement, GAD-7
13	Liang (27)	China	Patients with generalized anxiety disorder, 50–75 years old	4 weeks, 12 treatments (3 times per week)	Deqi sensation (soreness, numbness, distension, heaviness) required; 30-min needle retention, manipulation every 10 min	Sham acupuncture (no Deqi sensation)	70	SAS, SDS, PSQI
14	Lin (26)	China	Patients with generalized anxiety disorder	2 sessions per week, interval >48 h, 10 sessions total (5 weeks)	Deqi sensation (soreness, numbness, distension, heaviness) required; 30-min needle retention	Sham acupuncture (no Deqi sensation)	67	HAMA, SAS

### 3.3 Risk of bias

**Figure 2:**
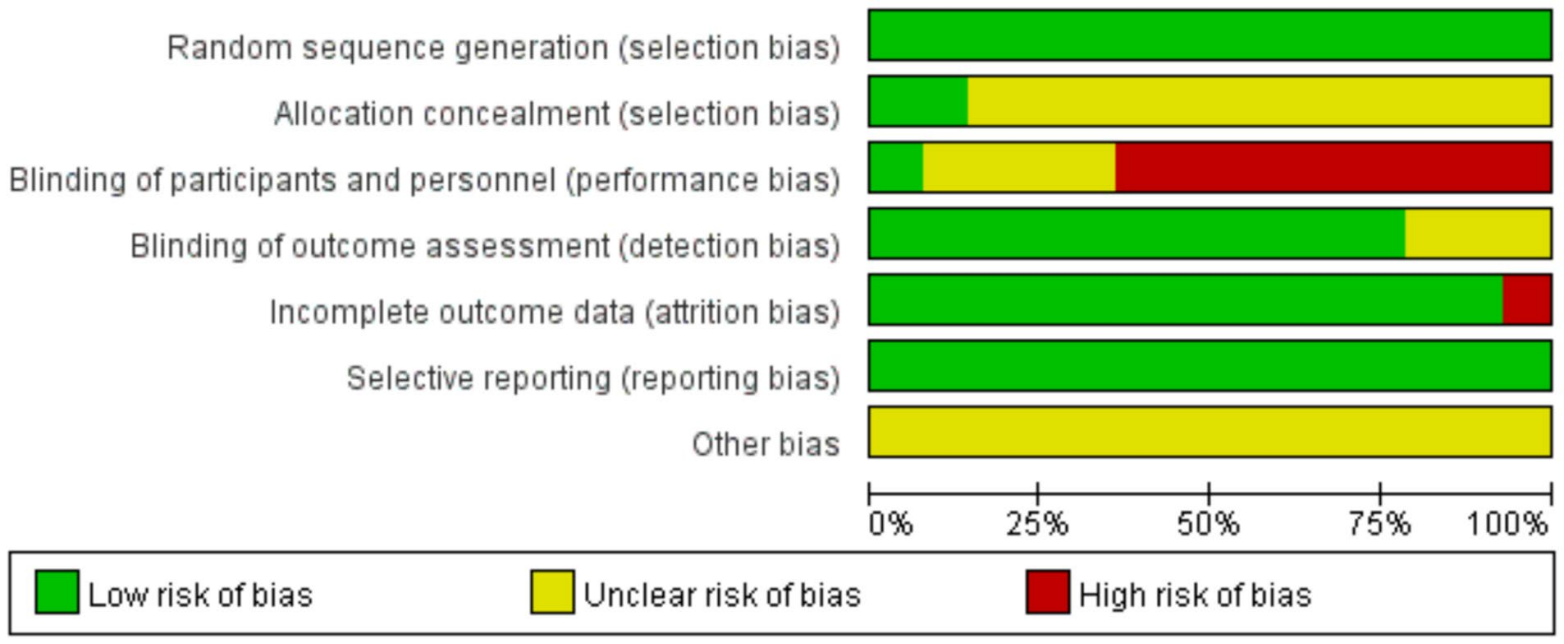
Graph of the risk of bias: percentage of all studies included.

**Figure 3:**
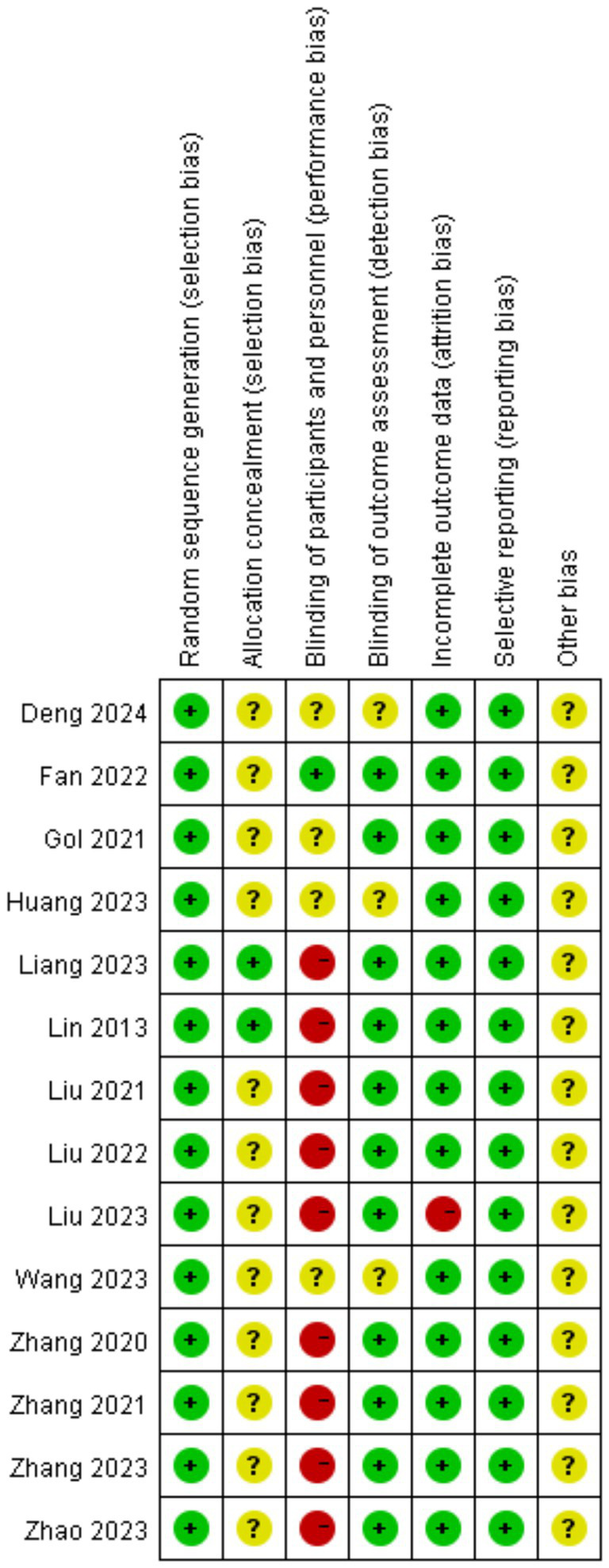
Risk of bias graph.

The methodological quality assessment of included studies, evaluated using the Cochrane Risk of Bias Tool, revealed mixed risk profiles across seven domains: random sequence generation and allocation concealment demonstrated predominantly low risk (75–100% and 50–75% of studies, respectively), indicating robust randomization practices, whereas performance bias (blinding of participants/personnel) and detection bias (blinding of outcome assessors) exhibited notable limitations, with 25–50% of studies rated as high risk due to inadequate blinding protocols inherent in acupuncture trials. Attrition and reporting biases were generally low (<25% high risk), supporting data completeness and transparency. Specific concerns included studies by Zhang et al. (17), showing high performance/detection bias risks, potentially inflating treatment effects, and Chinese-language studies [Liu (20) and Deng et al. (25)] lacking detailed blinding descriptions. These findings underscore the need for standardized blinding methodologies (e.g., non-penetrative sham devices) and explicit reporting in future trials to minimize placebo effects and enhance validity (Figures 2, 3).

### 3.4 Meta-analysis

#### 3.4.1 The HAMA score

**Figure 4:**
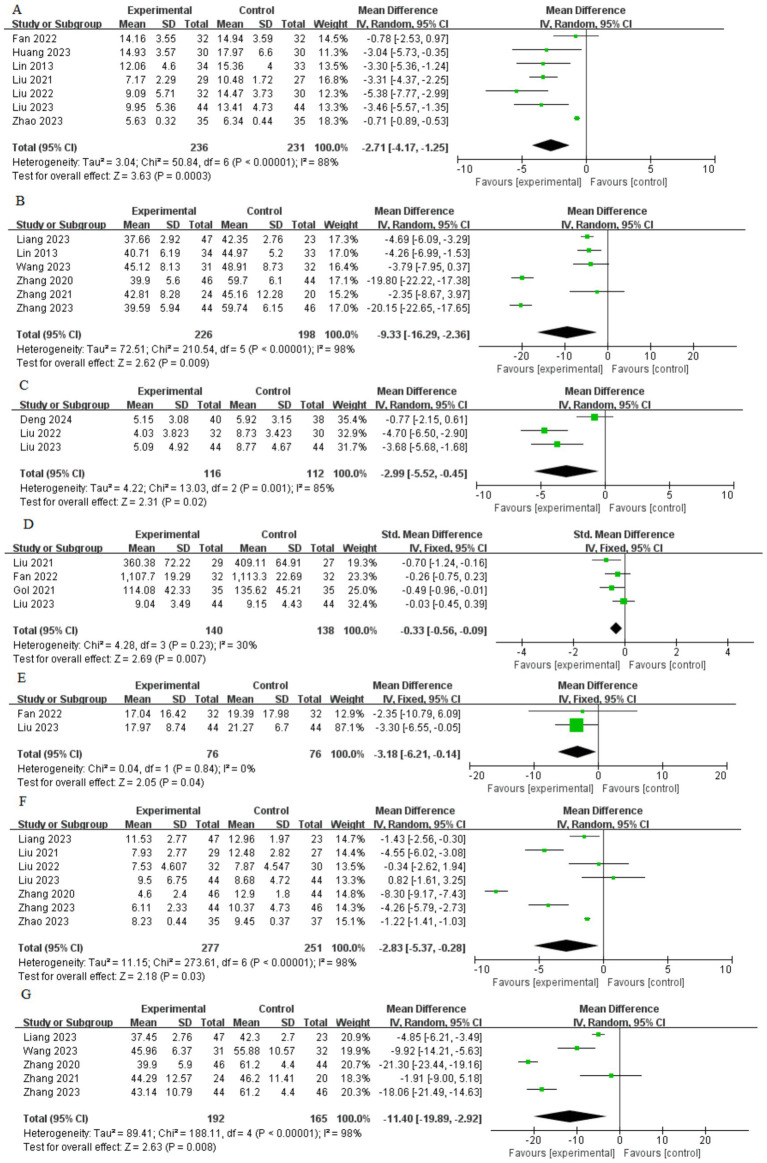
Forest plot of meta-analysis. **(A)** HAMA; **(B)** SAS; **(C)** GAD-7; **(D)** CORT; **(E)** ACTH; **(F)** PSQI; **(G)** SDS.

A total of seven articles were included to assess the reduction in the HAMA score. The studies exhibited significant heterogeneity (*I*^2^ = 88%, *p* < 0.00001), necessitating the application of a random-effects model (Figure 4A). Findings revealed that the HAMA score in the acupuncture group was significantly lower than in the control group [mean difference (MD) = −2.71, 95% confidence interval (CI) (−4.17, −1.25), *p* = 0.0003].

#### 3.4.2 The SAS score

Six articles presented data on the SAS score. A high level of heterogeneity across studies was observed (*I*^2^ = 98%, *p* < 0.0001), necessitating the use of the random-effects model (Figure 4B). Results revealed a statistically significant difference in clinical efficacy between the acupuncture group and the control group [MD = −9.33, 95% CI (−16.29, −2.36), *p* = 0.009].

#### 3.4.3 The GAD-7 score

Three studies reported data on the GAD-7 score. A high degree of heterogeneity was detected across the studies (*I*^2^ = 85%, *p* < 0.001), which required the application of a random-effects model (Figure 4C). The results demonstrated a statistically significant difference in clinical efficacy between the acupuncture group and the control group [MD = −2.99, 95% CI (−5.52, −0.45), *p* = 0.02].

#### 3.4.4 The CORT levels

Four studies provided data on the CORT score. No significant heterogeneity was observed among the studies, indicated by an *I*^2^ statistic of 30% (*p* = 0.23), necessitating the use of a fixed-effects model. The results did not indicate a statistically significant difference in clinical efficacy between the acupuncture and control groups, with an estimated standard standardized mean difference of [SMD = -0.33 (95% CI: −0.56, −0.09, *p* = 0.007)].

#### 3.4.5 The ACTH levels

Two studies contributed data on the ACTH score. No significant heterogeneity was noted across the studies, as evidenced by an *I*^2^ statistic of 0% (*p* = 0.84). This finding necessitated the application of a fixed-effects model (Figure 4E). The results yielded a statistically significant difference in clinical efficacy between the acupuncture group and the control group, with an estimated mean difference of −3.18 (95% CI: −6.21, −0.14; *p* = 0.04).

#### 3.4.6 The PSQI score

Seven studies presented data concerning the PSQI score, revealing significant heterogeneity among them as indicated by an *I*^2^ statistic of 98% (*p* < 0.00001), which justified the application of a random-effects model (Figure 4F). The analysis demonstrated a statistically significant difference in clinical efficacy between the acupuncture and control groups, reporting an estimated mean difference of −2.83 (95% CI: −5.37 to −0.28, *p* = 0.03).

#### 3.4.7 The SDS score

Five studies reported data on the SDS score. A high degree of heterogeneity was detected across the studies (*I*^2^ = 98%, *p* < 0.00001), which required the application of a random-effects model (Figure 4G). The results demonstrated a statistically significant difference in clinical efficacy between the acupuncture group and the control group [MD = −11.40, 95% CI (−19.89 to −2.92), *p* = 0.008].

### 3.5 Sensitivity analysis

Sensitivity analysis using the leave-one-out method was performed for all outcomes. The results indicated that the pooled effect sizes for all outcomes remained robust and were not excessively influenced by any single study, confirming the stability and reliability of our meta-analysis results (Supplementary material S2).

### 3.6 Publication bias

Because of the limited number of included studies, a funnel analysis was not conducted to evaluate publication bias. Therefore, Egger’s regression test and Begg’s rank correlation test were conducted for these outcomes (Table 2). Most outcomes showed no significant evidence of publication bias (*p* > 0.05), except for HAMA, where Egger’s test indicated potential small-study effects (*p* = 0.017), though Begg’s test was not significant (*p* = 0.548).

**Table 2:** Publication of biased results.

Outcome	Combination studies	Analysis model	Egger’s test	Begg’s test
HAMA	7	Random	0.017	0.548
SAS	6	Random	0.786	1.00
GAD-7	3	Random	0.306	1.00
CORT	4	Fixed	0.097	0.308
PSQI	7	Random	0.307	0.548
SDS	5	Random	0.624	0.806

### 3.7 Certainty of the evidence

The overall certainty of the evidence for each outcome, as assessed by the GRADE approach, is summarized in Table 3. The evidence certainty was rated as “low” for HAMA, SAS, and PSQI, due to serious risk of bias and serious inconsistency. The certainty for GAD-7 and SDS was “very low,” due to additional serious imprecision. The evidence for CORT and ACTH was rated as “moderate,” primarily downgraded for imprecision.

**Table 3:** GRADE evidence profile for the studies in the meta-analysis.

Outcome	No. study	No. patients	Certainty assessment					Summary of findings	
			Risk of bias	Inconsistency	Indirectness	Imprecision	Publication bias	MD/SMD (95%CI)	Certainty
HAMA	7	467	Serious	Serious	NS	NS	Serious	−2.17 (−4.17, −1.25)	Low
SAS	6	424	Serious	Serious	NS	NS	NS	−9.33 (−16.29, −2.36)	Low
GAD-7	3	228	Serious	Serious	NS	Serious	NS	−2.99 (−5.52, −0.45)	Very low
CORT	4	278	NS	NS	NS	Serious	NS	−0.33 (−0.56, −0.09)	Moderate
ACTH	2	152	NS	NS	NS	Serious	NS	−3.18 (−6.21, −0.14)	Moderate
PSQI	7	528	Serious	Serious	NS	NS	NS	−2.83 (−5.37, −0.28)	Low
SDS	5	357	Serious	Serious	NS	Serious	NS	−11.40 (−19.89, −2.92)	Very low

HAMA, the Hamilton Anxiety Scale; SAS, the Self-Rating Anxiety Scale; GAD-7, the Generalized Anxiety Disorder 7-item scale; CORT, Cortisol; ACTH, Adrenocorticotropic Hormone; PSQI, the Pittsburgh Sleep Quality Index; SDS, Self-Rating Depression Scale; No., number; CI, confidence interval; NS, not serious; MD, mean difference.

## 4 Discussion

This meta-analysis aimed to evaluate the comparative efficacy of acupuncture versus sham acupuncture for the treatment of generalized anxiety disorder (GAD). Our findings, based on 14 randomized controlled trials, support the hypothesis that acupuncture significantly reduces anxiety symptoms compared to sham acupuncture, with notable improvements observed across several patient-reported outcome measures, including the Hamilton Anxiety Scale (HAMA), the Self-Rating Anxiety Scale (SAS), the GAD-7 scale, the Self-Rating Depression Scale (SDS), and the Pittsburgh Sleep Quality Index (PSQI). The robustness of these findings was confirmed by sensitivity analysis. Despite the observed heterogeneity among studies, these results provide compelling evidence for the therapeutic potential of acupuncture in alleviating anxiety symptoms, which may be a valuable adjunct or alternative to traditional pharmacological treatments for GAD.

### 4.1 Efficacy of acupuncture in reducing anxiety symptoms

The primary outcome, HAMA, demonstrated a statistically significant reduction in anxiety symptoms for participants receiving acupuncture compared to the sham acupuncture group (MD = −2.71, 95% CI –4.17 to −1.25, *p* = 0.0003). This finding agrees with previous meta-analyses indicating that acupuncture may possess moderate efficacy in the management of GAD symptoms (8). When considering the clinical relevance of this finding, it is important to refer to the established minimal clinically important difference (MCID). For the HAMA scale in anxiety disorders, a reduction of 3 to 4 points is often considered clinically meaningful (28, 29). Our pooled estimate (MD = −2.71) is close to, though slightly below, this conventional threshold. This suggests that while acupuncture consistently outperforms sham acupuncture, the average effect across studies may represent a modest clinical benefit for the typical patient. However, it is crucial to note that a significant proportion of individuals within the studies likely experienced improvements meeting or exceeding the MCID, a nuance that underscores the therapy’s value despite the pooled mean falling just short of the benchmark.

The mechanism of action may involve the regulation of autonomic nervous system function (30) or the enhancement of inhibitory regulation within the limbic system, such as the amygdala (31–33). Acupuncture presents as a low-risk option for patients intolerant to medication or in need of prolonged treatment, especially for those who are sensitive to the potential adverse effects of medications (e.g., sedation, dependence) (34, 35). The high degree of heterogeneity observed across the included studies (*I*^2^ = 89%) is notable and indicates that variability in treatment protocols, such as acupuncture techniques, acupoint selection, and patient characteristics, may contribute to differences in effect sizes. While this variability warrants caution in generalizing the findings, the consistent pattern of improved outcomes across studies suggests that acupuncture exerts a beneficial effect that exceeds placebo responses.

### 4.2 Secondary outcomes: clinical interpretation of SAS, GAD-7, SDS, and PSQI

Secondary outcomes further corroborate the positive impact of acupuncture on anxiety and related domains. The improvement in the SAS score (MD = −9.33) is substantial. While a universally agreed-upon MCID for the SAS is less defined than for HAMA, a reduction of approximately 10 points is frequently cited in literature as indicative of meaningful clinical change (36). Our result, therefore, strongly suggests that the effect of acupuncture on self-reported anxiety is not only statistically significant but also clinically relevant. Similarly, for the GAD-7, a reduction of 2 to 3 points is considered the MCID (37). Our finding (MD = −2.99) meets this criterion, indicating a clinically meaningful reduction in generalized anxiety symptoms. The significant improvement in PSQI scores (MD = −2.83) is also noteworthy. The established MCID for the PSQI is typically around 3 points (38). Our result is very close to this threshold, suggesting that acupuncture may have a borderline clinically relevant effect on improving sleep quality in patients with GAD, a common and debilitating comorbidity.

In comparison to the meta-analysis of antidepressants (such as SSRIs) (39, 40), the effect size of acupuncture is slightly lower; however, its rate of adverse reactions is significantly lower (e.g., no withdrawal symptoms), indicating its advantages in the treatment of mild to moderate generalized anxiety disorder (GAD) or in maintenance therapy (41–43). Moreover, some studies (44–46) report that acupuncture can enhance 5-HT neurotransmission, which provides a theoretical basis for the “acupuncture combined with medication” strategy. Additionally, the SDS score, another important measure of the severity of anxiety and depression, also showed a significant improvement in the acupuncture group (MD = −11.4). These findings highlight the broad therapeutic effects of acupuncture across multiple facets of GAD, suggesting its potential role in addressing both the psychological and physiological components of the disorder. Interpreting the clinical significance of this change is complex due to the heterogeneity of MCID values reported for the SDS across different populations; however, reductions exceeding 10 points are generally regarded as substantial (47). This highlights the broad therapeutic effects of acupuncture across multiple facets of GAD, suggesting its potential role in addressing both the psychological and physiological components of the disorder.

### 4.3 Physiological outcomes and effects on the HPA axis

While acupuncture demonstrated consistent efficacy in reducing patient-reported anxiety symptoms, its impact on physiological biomarkers of the hypothalamic–pituitary–adrenal (HPA) axis presented a more complex picture. A significant reduction was observed in cortisol (CORT) levels favoring acupuncture (SMD = −0.33, 95% CI: −0.56 to −0.09, *p* = 0.007). However, it is important to interpret this finding in context. The effect size, as indicated by the standardized mean difference of −0.33, is considered ‘small’ according to conventional criteria (e.g., Cohen’s guidelines). This suggests that while a statistically genuine effect exists at the group level, the magnitude of the reduction in CORT may be modest from a clinical or physiological perspective.

Interestingly, a significant reduction was also found in ACTH levels (MD = −3.18, 95% CI –6.21 to −0.14, *p* = 0.04). The concurrent, albeit modest, reduction in both ACTH and CORT provides preliminary evidence that acupuncture may exert a modulating influence on the HPA axis, potentially acting at or above the level of the pituitary to attenuate the stress response (48). The smaller effect size for CORT compared to the more pronounced effects on self-reported anxiety could indicate that: (1) the primary mechanisms of acupuncture for GAD involve central nervous system pathways [e.g., limbic system modulation (31–33)] that are not fully reflected in peripheral cortisol levels; (2) the relationship between psychological symptom improvement and HPA axis normalization is not linear and may be influenced by factors such as individual variability, chronicity of stress, or the timing of biomarker measurement (49); or (3) sham acupuncture itself may have some physiological effects, thereby reducing the observed difference between groups.

### 4.4 Implications for clinical practice

Our findings suggest that acupuncture could serve as a valuable adjunctive or alternative treatment for GAD, particularly for patients seeking non-pharmacological interventions, those who have not responded well to conventional treatments, or those concerned about medication side effects. The clinically meaningful improvements observed in key patient-reported outcomes like the SAS, GAD-7, and PSQI provide a strong evidence base for recommending acupuncture in clinical practice. However, clinicians should be mindful of the substantial heterogeneity across studies, which may be attributed to differences in acupuncture techniques, treatment durations, and follow-up periods. To maximize the clinical benefits of acupuncture, further research is needed to establish optimal and standardized acupuncture protocols, including frequency, duration, and selection of acupoints.

### 4.5 Limitations and future research

Several limitations must be acknowledged in this meta-analysis. First, the majority of the studies included were conducted in China, which may limit the generalizability of the results to other populations. Additionally, the heterogeneity observed across studies complicates the interpretation of pooled effect sizes. Variations in acupuncture techniques, treatment durations, and follow-up periods introduce significant variability that could affect the outcomes. Moreover, methodological quality concerns, particularly around performance bias and detection bias due to inadequate blinding, suggest that the current evidence is not without limitations.

Future research should focus on conducting high-quality, multi-center trials with rigorous blinding protocols to minimize potential biases. Furthermore, future trials should be designed to specifically assess the proportion of patients achieving clinically significant improvement (e.g., using MCID thresholds or responder analyses), which will provide a clearer picture of acupuncture’s real-world applicability. Standardized acupuncture protocols should be developed to reduce variability in treatment and ensure more consistent results. Additionally, further studies are needed to explore the physiological mechanisms of acupuncture in GAD patients, particularly with respect to its effects on cortisol, ACTH, and other biomarkers of stress. The role of acupuncture in sleep improvement also warrants further exploration, as sleep disturbances are a common comorbidity of GAD.

## 5 Conclusion

In conclusion, acupuncture appears to be an effective treatment for generalized anxiety disorder, demonstrating significant improvements in anxiety symptoms compared to sham acupuncture. While the variability in treatment protocols and study methodologies calls for caution in the interpretation of the results, acupuncture offers promise as a complementary therapeutic option for GAD. Further research is necessary to optimize treatment protocols, address methodological weaknesses, and deepen our understanding of the physiological mechanisms underlying acupuncture’s effects on anxiety.

## Data Availability

The original contributions presented in the study are included in the article/Supplementary material, further inquiries can be directed to the corresponding authors.

## Footnotes

^1^ https://www.clinicaltrials.gov/
