# Supplementary material for: Efficacy of acupuncture versus sham acupuncture on generalized anxiety disorder: a meta-analysis of randomized controlled trials
Source: Front Neurol. 2025 Nov 12;16:1682400. doi: 10.3389/fneur.2025.1682400 (PMC12646924; doi:10.3389/fneur.2025.1682400)
Supplement: Supplementary file 2 [file Table_2.docx]

PubMed search strategy

#1 “Anxiety Disorders”[Mesh] OR “Generalized Anxiety Disorder”[Mesh]

#2 (generalized anxiety[tiab]) OR (GAD[tiab]) OR (anxiety neurosis[tiab]) OR (Anxiety State[tiab]) OR (Anxiety Neuroses[tiab])

#3 #1 OR #2

#4 “Acupuncture Therapy”[Mesh] OR “Acupuncture”[Mesh]

#5 (acupunct*[tiab]) OR (electroacupuncture[tiab]) OR (meridian therapy[tiab]) OR (Acupuncture Treatment[tiab])

#6 #4 OR #5

#7 “Randomized Controlled Trial”[tiab] OR randomized[tiab] OR randomised[tiab] OR RCT[tiab]

#8 #3 AND #6 AND #7
