# Supplementary material for: Efficacy of acupuncture versus sham acupuncture on generalized anxiety disorder: a meta-analysis of randomized controlled trials
Source: Front Neurol. 2025 Nov 12;16:1682400. doi: 10.3389/fneur.2025.1682400 (PMC12646924; doi:10.3389/fneur.2025.1682400)
Supplement: Supplementary file 3 [file Table_3.docx]

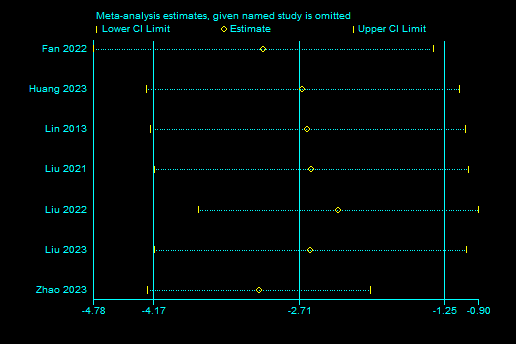


HAMA sensitivity analysis results


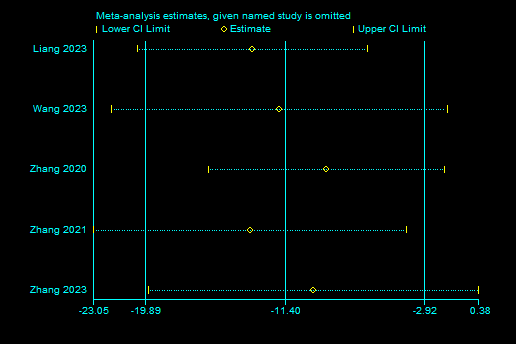


SDS sensitivity analysis results


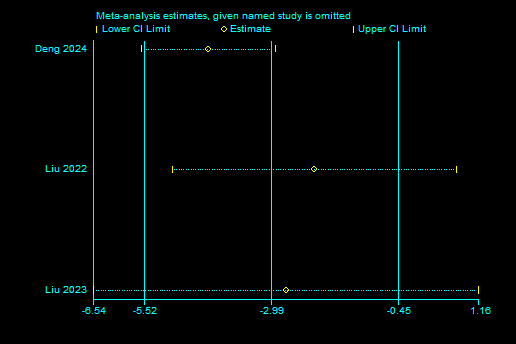
GAD-7 sensitivity analysis results


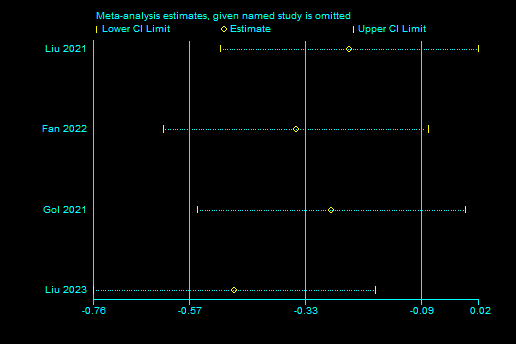


CORT sensitivity analysis results


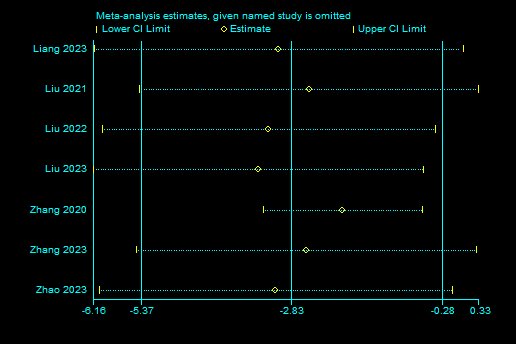


PSQI sensitivity analysis results


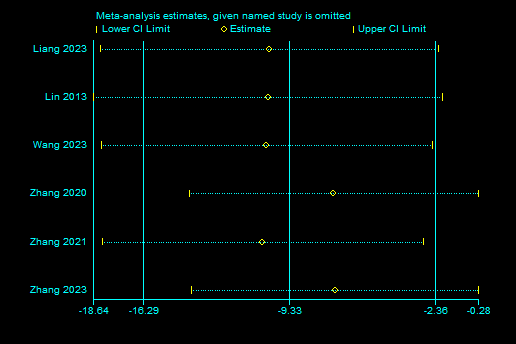


SAS sensitivity analysis results
